# 1-{[4′-(1*H*-1,2,4-Triazol-2-ium-1-ylmeth­yl)biphenyl-4-yl]meth­yl}-1*H*-1,2,4-triazol-2-ium bis­(3-carb­oxy-5-iodo­benzoate)–5-iodo­benzene-3,5-dicarb­oxy­lic acid–water (1/2/2)

**DOI:** 10.1107/S1600536812014584

**Published:** 2012-04-13

**Authors:** Kou-Lin Zhang, Ye Deng, Seik Weng Ng

**Affiliations:** a College of Chemistry and Chemical Engineering, Yangzhou University, Yangzhou 225002, People’s Republic of China; b Department of Chemistry, University of Malaya, 50603 Kuala Lumpur, Malaysia; c Chemistry Department, King Abdulaziz University, PO Box 80203 Jeddah, Saudi Arabia

## Abstract

The neutral carb­oxy­lic acid mol­ecule and the carboxyl­ate anion in the title compound, C_18_H_18_N_6_^2+^·2C_8_H_4_IO_4_^−^·2C_8_H_5_IO_4_·2H_2_O, are both nearly planar (r.m.s. deviations = 0.034 and 0.045 Å, respectively). In the cation, the mid-point of the C—C bond linking the two benzene rings lies on a center of inversion, and the triazole ring is approximately perpendicular to the adjacent benzene ring [dihedral angle = 83.2 (3)°]. In the crystal, the cations, anions, carb­oxy­lic acid and lattice water mol­ecules are linked by N—H⋯O, O—H⋯N and O—H⋯O hydrogen bonds, generating a ribbon running along [1-10]. The crystal studied was a non-merohedral twin with the components in a 51.2 (1):48.8 (1) ratio.

## Related literature

For the structure of 5-iodo­isophthalic acid, see: Zang *et al.* (2011 ▶).
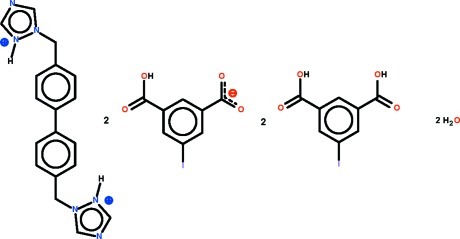


## Experimental

### 

#### Crystal data

C_18_H_18_N_6_^2+^·2C_8_H_4_IO_4_^−^·2C_8_H_5_IO_4_·2H_2_O
*M* *_r_* = 1520.48
Triclinic, 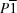

*a* = 8.2620 (9) Å
*b* = 9.6859 (10) Å
*c* = 18.661 (2) Å
α = 85.413 (1)°
β = 89.262 (1)°
γ = 65.084 (1)°
*V* = 1349.7 (2) Å^3^
*Z* = 1
Mo *K*α radiation
μ = 2.39 mm^−1^
*T* = 293 K
0.25 × 0.20 × 0.15 mm

#### Data collection

Bruker SMART APEX diffractometer
Absorption correction: multi-scan (TWINABS; Bruker, 2005 ▶) *T*_min_ = 0.576, *T*_max_ = 0.746
17086 measured reflections
6404 independent reflections
4887 reflections with *I* > 2σ(*I*)
*R*_int_ = 0.046

#### Refinement

*R*[*F* ^2^ > 2σ(*F* ^2^)] = 0.041
*wR*(*F* ^2^) = 0.116
*S* = 1.07
6402 reflections
356 parameters
3 restraints
H-atom parameters constrained
Δρ_max_ = 0.51 e Å^−3^
Δρ_min_ = −1.03 e Å^−3^

### 

Data collection: *APEX2* (Bruker, 2005 ▶); cell refinement: *SAINT* (Bruker, 2005 ▶); data reduction: *SAINT*; program(s) used to solve structure: *SHELXS97* (Sheldrick, 2008 ▶); program(s) used to refine structure: *SHELXL97* (Sheldrick, 2008 ▶); molecular graphics: *X-SEED* (Barbour, 2001 ▶); software used to prepare material for publication: *publCIF* (Westrip, 2010 ▶).

## Supplementary Material

Crystal structure: contains datablock(s) global, I. DOI: 10.1107/S1600536812014584/xu5507sup1.cif

Structure factors: contains datablock(s) I. DOI: 10.1107/S1600536812014584/xu5507Isup2.hkl

Additional supplementary materials:  crystallographic information; 3D view; checkCIF report

## Figures and Tables

**Table 1:** Hydrogen-bond geometry (Å, °)

*D*—H⋯*A*	*D*—H	H⋯*A*	*D*⋯*A*	*D*—H⋯*A*
O1—H1⋯O4^i^	0.84	1.90	2.666 (6)	150
O3—H2⋯N3	0.84	1.84	2.642 (6)	159
O5—H3⋯O8^ii^	0.84	1.93	2.628 (6)	140
O1w—H5⋯O6^i^	0.84	1.97	2.803 (7)	172
O1w—H6⋯O7	0.84	1.81	2.638 (7)	171
N2—H4⋯O1w	0.88	2.12	2.899 (6)	148

Symmetry codes: (i) 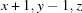; (ii) 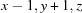.

**Fig. 1.**
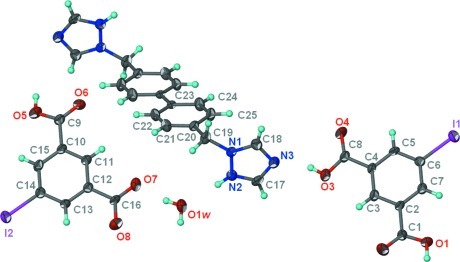
Thermal ellipsoid plot (Barbour, 2001) of (C18H18N6)2+ 2(C8H4IO4)-.2C8H5IO4.2H2O at the 50% probability level; hydrogen atoms are drawn as spheres of arbitrary radius. The cation lies on a center-of-inversion and symmetry-related atoms are not labeled.

**Fig. 2.**
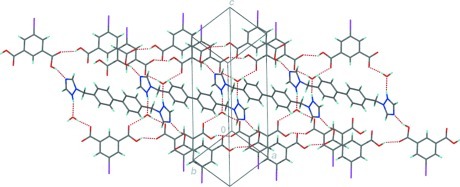
Hydrogen-bond ribbon structure.

## supplementary crystallographic information

### Comment

5-Iodoisophthalic acid furnishes a number of coordination polymers; these feature iodine···π interactions (Zang *et al.*, 2011). Our attempt at synthesizing the cadmium derivative, which was expected to be further connected through 4,4'-bis(1,2,4-triazol-1-ylmethyl)biphenyl, returned instead the salt,(C_18_H_18_N_6_)^2+^ 2(C_8_H_4_IO_4_)^-.^2C_8_H_5_IO_4_^.^2H_2_O (Scheme I, Fig. 1). The carboxylate ion and the neutral carboxylic acid are both planar (r.m.s.deviation 0.034 and 0.045 Å). The mid-point of the C_phenylene_–C_phenylene_ bond lies on a center-of-inversion. The cation, anion, carboxylic acid and water molecules are linked by N–H···O and O–H···O hydrogen bonds to generate a ribbon running along [1 - 1 0] (Table 1, Fig. 2).

### Experimental

A mixture containing cadmium chloride (12.8 mg, 0.1 mmol), 5-iodoisophthalic acid (29.0 mg, 0.1 mmol), 4,4'-bis(1,2,4-triazol-1-ylmethyl)biphenyl (31.6 mg, 0.1 mmol), water (6 ml) and perchloric acid (1 drop) was sealed in a 23 ml, Teflon-lined, stainless-steel Parr bomb. This was heated at 393 K for 3 days. Yellow crystals were isolated from the vessel.

### Refinement

Carbon-bound H-atoms were placed in calculated positions (C–H 0.93 Å) and were included in the refinement in the riding model approximation, with *U*(H) set to 1.2*U*(C).

For the carboxyl groups, an acid hydrogen was placed on the oxygen atom with long C–O bonds, *i.e.*, *ca*. 1.30 Å in the riding mode. One carboxylic entity has one such long bonds whereas the other has two; in the latter case, the entity was assumed to be a neutral carboxylic acid molecule. The water hydrogen atoms were placed in chemically sensible positions on the basis of hydrogen-bonding interactions; the O–H distance was set to 0.84 Å, and the temperature factors were set to 1.5 times that of the parent atom. In this scheme of hydrogen atoms, the nitrogen atom at the 2-position of the triazole should be protonated; this was treated as riding [N–H = 0.88 Å, *U*(H) = 1.2*U*(N)].

The final difference Fourier map had a peak at 0.73 Å from I1.

Omitted owing to bad disagreement were (6 4 4), (8 3 7), (-4 - 5 2), (5 4 0), (-3 - 2 14), (-2 8 0), (1 - 2 3), (2 1 1), (-6 - 4 3), (9 3 7), (7 4 4),(-1 8 0), (-2 - 1 1), (8 4 8), (-7 4 7), (-4 - 6 3), (-3 - 6 3), (-6 4 7), (-5 4 7) and (8 3 10). The large number of omissions is an artifact of twinning. The crystal is a non-merohedral twin with the components being in a 51.2 (1): 48.8 ratio.

### Crystal data

**Table:**

C_18_H_18_N_6_^2+^·2C_8_H_4_IO_4_^−^·2C_8_H_5_IO_4_·2H_2_O	*Z* = 1
*M**_r_* = 1520.48	*F*(000) = 738
Triclinic, *P*1	*D*_x_ = 1.871 Mg m^−^^3^
Hall symbol: -P 1	Mo *K*α radiation, λ = 0.71073 Å
*a* = 8.2620 (9) Å	Cell parameters from 1261 reflections
*b* = 9.6859 (10) Å	θ = 3.3–23.5°
*c* = 18.661 (2) Å	µ = 2.39 mm^−^^1^
α = 85.413 (1)°	*T* = 293 K
β = 89.262 (1)°	Prism, yellow
γ = 65.084 (1)°	0.25 × 0.20 × 0.15 mm
*V* = 1349.7 (2) Å^3^	

### Data collection

**Table:**

Bruker SMART APEX diffractometer	6404 independent reflections
Radiation source: fine-focus sealed tube	4887 reflections with *I* > 2σ(*I*)
Graphite monochromator	*R*_int_ = 0.046
ω scans	θ_max_ = 28.3°, θ_min_ = 2.2°
Absorption correction: multi-scan (TWINABS; Bruker, 2005)	*h* = −10→10
*T*_min_ = 0.576, *T*_max_ = 0.746	*k* = −12→12
17086 measured reflections	*l* = 0→24

### Refinement

**Table:**

Refinement on *F*^2^	Primary atom site location: structure-invariant direct methods
Least-squares matrix: full	Secondary atom site location: difference Fourier map
*R*[*F*^2^ > 2σ(*F*^2^)] = 0.041	Hydrogen site location: inferred from neighbouring sites
*wR*(*F*^2^) = 0.116	H-atom parameters constrained
*S* = 1.07	*w* = 1/[σ^2^(*F*_o_^2^) + (0.0542*P*)^2^ + 0.3162*P*] where *P* = (*F*_o_^2^ + 2*F*_c_^2^)/3
6402 reflections	(Δ/σ)_max_ = 0.001
356 parameters	Δρ_max_ = 0.51 e Å^−^^3^
3 restraints	Δρ_min_ = −1.03 e Å^−^^3^

### Fractional atomic coordinates and isotropic or equivalent isotropic displacement parameters (Å^2^)

**Table:**

	*x*	*y*	*z*	*U*_iso_*/*U*_eq_	
I1	1.50742 (6)	0.26356 (5)	−0.014572 (19)	0.04613 (13)	
I2	−0.01012 (6)	0.73309 (5)	1.025823 (19)	0.04621 (13)	
O1	1.7771 (7)	−0.2217 (5)	0.1873 (2)	0.0617 (14)	
H1	1.8440	−0.3039	0.2104	0.093*	
O2	1.6412 (8)	−0.1715 (6)	0.2909 (3)	0.090 (2)	
O3	1.1179 (6)	0.3175 (5)	0.3034 (2)	0.0545 (12)	
H2	1.0302	0.3831	0.3230	0.082*	
O4	1.0457 (7)	0.5034 (5)	0.2147 (3)	0.0670 (15)	
O5	−0.2756 (7)	1.2072 (6)	0.8193 (3)	0.0674 (15)	
H3	−0.3436	1.2867	0.7946	0.101*	
O6	−0.1152 (7)	1.1631 (5)	0.7186 (2)	0.0629 (15)	
O7	0.3845 (7)	0.6420 (6)	0.7091 (3)	0.0642 (14)	
O8	0.4582 (7)	0.4805 (6)	0.8062 (3)	0.0673 (15)	
O1W	0.6642 (8)	0.4440 (6)	0.6460 (3)	0.0882 (19)	
H5	0.7237	0.3629	0.6714	0.132*	
H6	0.5784	0.5002	0.6700	0.132*	
N1	0.7596 (6)	0.6294 (5)	0.4816 (2)	0.0325 (10)	
N2	0.7346 (6)	0.5001 (5)	0.4964 (2)	0.0466 (14)	
H4	0.6795	0.4775	0.5330	0.056*	
N3	0.8857 (7)	0.4846 (6)	0.3953 (3)	0.0471 (13)	
C1	1.6570 (8)	−0.1375 (7)	0.2290 (3)	0.0453 (15)	
C2	1.5356 (7)	0.0170 (6)	0.1926 (3)	0.0336 (12)	
C3	1.3960 (7)	0.1186 (6)	0.2312 (3)	0.0358 (12)	
H2A	1.3745	0.0900	0.2779	0.043*	
C4	1.2904 (8)	0.2622 (6)	0.1992 (3)	0.0341 (12)	
C5	1.3195 (7)	0.3059 (6)	0.1289 (3)	0.0358 (12)	
H5A	1.2481	0.4030	0.1081	0.043*	
C6	1.4566 (7)	0.2024 (6)	0.0901 (3)	0.0328 (12)	
C7	1.5642 (7)	0.0595 (7)	0.1226 (3)	0.0358 (12)	
H7	1.6568	−0.0089	0.0970	0.043*	
C8	1.1387 (8)	0.3744 (7)	0.2392 (3)	0.0395 (13)	
C9	−0.1449 (8)	1.1247 (7)	0.7801 (3)	0.0407 (14)	
C10	−0.0290 (8)	0.9701 (6)	0.8159 (3)	0.0370 (13)	
C11	0.1075 (7)	0.8649 (6)	0.7795 (3)	0.0352 (12)	
H11	0.1308	0.8900	0.7325	0.042*	
C12	0.2110 (7)	0.7203 (6)	0.8133 (3)	0.0338 (12)	
C13	0.1764 (7)	0.6829 (6)	0.8832 (3)	0.0352 (12)	
H13	0.2443	0.5861	0.9052	0.042*	
C14	0.0413 (7)	0.7890 (7)	0.9203 (3)	0.0371 (12)	
C15	−0.0623 (7)	0.9314 (7)	0.8872 (3)	0.0369 (12)	
H15	−0.1543	1.0022	0.9120	0.044*	
C16	0.3617 (8)	0.6048 (6)	0.7742 (3)	0.0390 (13)	
C17	0.8114 (8)	0.4178 (7)	0.4436 (4)	0.0462 (15)	
H17	0.8149	0.3215	0.4394	0.055*	
C18	0.8480 (7)	0.6189 (7)	0.4213 (3)	0.0412 (14)	
H18	0.8793	0.6941	0.4001	0.049*	
C19	0.6785 (8)	0.7581 (7)	0.5267 (3)	0.0417 (14)	
H19A	0.7250	0.8332	0.5137	0.050*	
H19B	0.7104	0.7219	0.5768	0.050*	
C20	0.4761 (8)	0.8326 (6)	0.5174 (3)	0.0324 (11)	
C21	0.3719 (8)	0.8896 (7)	0.5758 (3)	0.0393 (13)	
H21	0.4256	0.8836	0.6201	0.047*	
C22	0.1836 (7)	0.9571 (7)	0.5684 (3)	0.0379 (13)	
H22	0.1152	0.9953	0.6082	0.046*	
C23	0.0993 (6)	0.9677 (6)	0.5040 (3)	0.0274 (11)	
C24	0.2069 (8)	0.9138 (7)	0.4446 (3)	0.0441 (14)	
H24	0.1531	0.9215	0.4000	0.053*	
C25	0.3932 (7)	0.8488 (7)	0.4509 (3)	0.0422 (14)	
H25	0.4619	0.8162	0.4105	0.051*	

### Atomic displacement parameters (Å^2^)

**Table:**

	*U*^11^	*U*^22^	*U*^33^	*U*^12^	*U*^13^	*U*^23^
I1	0.0417 (2)	0.0500 (3)	0.03632 (19)	−0.01081 (19)	0.00665 (19)	0.00430 (19)
I2	0.0433 (2)	0.0489 (3)	0.03718 (19)	−0.01146 (19)	0.00968 (19)	0.00070 (19)
O1	0.062 (3)	0.036 (3)	0.046 (3)	0.018 (2)	0.005 (2)	0.009 (2)
O2	0.096 (4)	0.060 (3)	0.046 (3)	0.027 (3)	0.019 (3)	0.021 (2)
O3	0.054 (3)	0.046 (3)	0.038 (2)	0.003 (2)	0.010 (2)	−0.005 (2)
O4	0.059 (3)	0.039 (3)	0.058 (3)	0.021 (2)	0.019 (2)	0.001 (2)
O5	0.059 (3)	0.042 (3)	0.060 (3)	0.018 (2)	0.009 (3)	0.003 (2)
O6	0.080 (4)	0.038 (3)	0.036 (3)	0.006 (3)	−0.003 (2)	0.0080 (19)
O7	0.075 (4)	0.052 (3)	0.044 (3)	−0.006 (3)	0.021 (2)	−0.008 (2)
O8	0.061 (3)	0.042 (3)	0.066 (3)	0.010 (2)	0.013 (3)	0.001 (2)
O1W	0.124 (5)	0.056 (3)	0.048 (3)	−0.003 (3)	0.041 (3)	−0.003 (2)
N1	0.028 (2)	0.029 (3)	0.035 (2)	−0.006 (2)	0.0020 (18)	−0.0018 (18)
N2	0.056 (4)	0.035 (3)	0.047 (3)	−0.019 (3)	0.022 (3)	−0.002 (2)
N3	0.040 (3)	0.039 (3)	0.042 (3)	0.003 (2)	0.007 (2)	−0.002 (2)
C1	0.045 (4)	0.037 (3)	0.031 (3)	0.004 (3)	0.005 (3)	0.002 (2)
C2	0.031 (3)	0.025 (3)	0.032 (3)	0.001 (2)	0.002 (2)	−0.004 (2)
C3	0.031 (3)	0.037 (3)	0.031 (3)	−0.006 (3)	0.004 (2)	−0.007 (2)
C4	0.035 (3)	0.029 (3)	0.029 (3)	−0.004 (2)	0.006 (2)	−0.008 (2)
C5	0.037 (3)	0.020 (3)	0.037 (3)	−0.001 (2)	0.000 (2)	0.000 (2)
C6	0.029 (3)	0.032 (3)	0.033 (3)	−0.009 (2)	0.000 (2)	0.002 (2)
C7	0.027 (3)	0.037 (3)	0.034 (3)	−0.004 (2)	0.005 (2)	−0.005 (2)
C8	0.033 (3)	0.035 (3)	0.036 (3)	0.000 (3)	0.001 (2)	−0.008 (2)
C9	0.044 (4)	0.032 (3)	0.034 (3)	−0.003 (3)	−0.004 (2)	−0.005 (2)
C10	0.033 (3)	0.027 (3)	0.035 (3)	0.002 (2)	0.001 (2)	−0.003 (2)
C11	0.028 (3)	0.030 (3)	0.036 (3)	−0.001 (2)	0.002 (2)	−0.002 (2)
C12	0.032 (3)	0.031 (3)	0.032 (3)	−0.006 (2)	0.000 (2)	−0.004 (2)
C13	0.031 (3)	0.026 (3)	0.033 (3)	0.002 (2)	−0.001 (2)	0.003 (2)
C14	0.034 (3)	0.037 (3)	0.034 (3)	−0.008 (3)	0.004 (2)	−0.003 (2)
C15	0.028 (3)	0.035 (3)	0.041 (3)	−0.005 (2)	0.006 (2)	−0.010 (2)
C16	0.037 (3)	0.023 (3)	0.042 (3)	0.002 (3)	0.004 (2)	−0.004 (2)
C17	0.047 (4)	0.025 (3)	0.055 (4)	−0.004 (3)	0.009 (3)	−0.006 (3)
C18	0.038 (3)	0.042 (4)	0.041 (3)	−0.015 (3)	0.009 (2)	0.001 (3)
C19	0.036 (3)	0.041 (4)	0.045 (3)	−0.011 (3)	0.006 (3)	−0.012 (3)
C20	0.031 (3)	0.027 (3)	0.036 (2)	−0.010 (2)	0.005 (3)	−0.007 (2)
C21	0.039 (3)	0.051 (4)	0.030 (3)	−0.020 (3)	0.001 (2)	−0.005 (2)
C22	0.033 (3)	0.048 (4)	0.031 (3)	−0.015 (3)	0.009 (2)	−0.004 (2)
C23	0.027 (3)	0.021 (3)	0.028 (2)	−0.004 (2)	0.002 (2)	0.0001 (19)
C24	0.041 (4)	0.057 (4)	0.027 (3)	−0.013 (3)	−0.004 (2)	−0.006 (3)
C25	0.032 (3)	0.049 (4)	0.035 (3)	−0.005 (3)	0.006 (2)	−0.011 (3)

### Geometric parameters (Å, º)

**Table:**

I1—C6	2.085 (5)	C5—H5A	0.9300
I2—C14	2.086 (5)	C6—C7	1.384 (7)
O1—C1	1.287 (7)	C7—H7	0.9300
O1—H1	0.8400	C9—C10	1.502 (8)
O2—C1	1.195 (8)	C10—C11	1.376 (8)
O3—C8	1.318 (7)	C10—C15	1.408 (7)
O3—H2	0.8400	C11—C12	1.398 (7)
O4—C8	1.212 (7)	C11—H11	0.9300
O5—C9	1.298 (7)	C12—C13	1.385 (7)
O5—H3	0.8400	C12—C16	1.506 (8)
O6—C9	1.232 (7)	C13—C14	1.382 (7)
O7—C16	1.273 (7)	C13—H13	0.9300
O8—C16	1.237 (7)	C14—C15	1.378 (8)
O1W—H5	0.8400	C15—H15	0.9300
O1W—H6	0.8400	C17—H17	0.9300
N1—C18	1.321 (6)	C18—H18	0.9300
N1—N2	1.359 (6)	C19—C20	1.523 (8)
N1—C19	1.469 (7)	C19—H19A	0.9700
N2—C17	1.302 (7)	C19—H19B	0.9700
N2—H4	0.8800	C20—C21	1.380 (7)
N3—C18	1.335 (8)	C20—C25	1.391 (7)
N3—C17	1.355 (7)	C21—C22	1.414 (7)
C1—C2	1.515 (8)	C21—H21	0.9300
C2—C7	1.387 (7)	C22—C23	1.371 (7)
C2—C3	1.397 (7)	C22—H22	0.9300
C3—C4	1.382 (8)	C23—C24	1.404 (7)
C3—H2A	0.9300	C23—C23^i^	1.495 (10)
C4—C5	1.395 (7)	C24—C25	1.399 (7)
C4—C8	1.505 (7)	C24—H24	0.9300
C5—C6	1.397 (7)	C25—H25	0.9300
			
C1—O1—H1	109.5	C13—C12—C16	119.6 (5)
C8—O3—H2	109.5	C11—C12—C16	120.3 (5)
C9—O5—H3	109.5	C14—C13—C12	120.3 (5)
H5—O1W—H6	108.6	C14—C13—H13	119.9
C18—N1—N2	109.3 (5)	C12—C13—H13	119.9
C18—N1—C19	130.2 (5)	C15—C14—C13	120.0 (5)
N2—N1—C19	120.3 (4)	C15—C14—I2	119.7 (4)
C17—N2—N1	103.6 (4)	C13—C14—I2	120.3 (4)
C17—N2—H4	128.2	C14—C15—C10	120.1 (5)
N1—N2—H4	128.2	C14—C15—H15	119.9
C18—N3—C17	102.9 (5)	C10—C15—H15	119.9
O2—C1—O1	125.4 (6)	O8—C16—O7	123.1 (5)
O2—C1—C2	121.5 (6)	O8—C16—C12	119.4 (5)
O1—C1—C2	113.1 (5)	O7—C16—C12	117.5 (5)
C7—C2—C3	120.0 (5)	N2—C17—N3	114.1 (5)
C7—C2—C1	120.9 (5)	N2—C17—H17	123.0
C3—C2—C1	119.2 (5)	N3—C17—H17	123.0
C4—C3—C2	119.2 (5)	N1—C18—N3	110.1 (5)
C4—C3—H2A	120.4	N1—C18—H18	125.0
C2—C3—H2A	120.4	N3—C18—H18	125.0
C3—C4—C5	121.0 (5)	N1—C19—C20	111.1 (5)
C3—C4—C8	120.6 (5)	N1—C19—H19A	109.4
C5—C4—C8	118.4 (5)	C20—C19—H19A	109.4
C4—C5—C6	119.4 (5)	N1—C19—H19B	109.4
C4—C5—H5A	120.3	C20—C19—H19B	109.4
C6—C5—H5A	120.3	H19A—C19—H19B	108.0
C7—C6—C5	119.5 (5)	C21—C20—C25	119.0 (5)
C7—C6—I1	119.6 (4)	C21—C20—C19	119.4 (5)
C5—C6—I1	120.8 (4)	C25—C20—C19	121.6 (5)
C6—C7—C2	120.8 (5)	C20—C21—C22	120.2 (5)
C6—C7—H7	119.6	C20—C21—H21	119.9
C2—C7—H7	119.6	C22—C21—H21	119.9
O4—C8—O3	123.7 (5)	C23—C22—C21	121.7 (5)
O4—C8—C4	123.4 (5)	C23—C22—H22	119.2
O3—C8—C4	112.9 (5)	C21—C22—H22	119.2
O6—C9—O5	125.4 (6)	C22—C23—C24	117.5 (5)
O6—C9—C10	120.9 (5)	C22—C23—C23^i^	122.4 (6)
O5—C9—C10	113.7 (5)	C24—C23—C23^i^	120.0 (6)
C11—C10—C15	119.7 (5)	C25—C24—C23	121.4 (5)
C11—C10—C9	120.7 (5)	C25—C24—H24	119.3
C15—C10—C9	119.6 (5)	C23—C24—H24	119.3
C10—C11—C12	119.8 (5)	C20—C25—C24	120.1 (5)
C10—C11—H11	120.1	C20—C25—H25	119.9
C12—C11—H11	120.1	C24—C25—H25	119.9
C13—C12—C11	120.1 (5)		
			
C18—N1—N2—C17	0.4 (6)	C16—C12—C13—C14	178.4 (5)
C19—N1—N2—C17	175.2 (5)	C12—C13—C14—C15	1.5 (9)
O2—C1—C2—C7	174.9 (7)	C12—C13—C14—I2	−179.7 (4)
O1—C1—C2—C7	−3.3 (9)	C13—C14—C15—C10	−1.0 (9)
O2—C1—C2—C3	−3.5 (10)	I2—C14—C15—C10	−179.8 (4)
O1—C1—C2—C3	178.3 (6)	C11—C10—C15—C14	−0.1 (9)
C7—C2—C3—C4	−1.4 (9)	C9—C10—C15—C14	178.9 (5)
C1—C2—C3—C4	177.0 (6)	C13—C12—C16—O8	−3.4 (9)
C2—C3—C4—C5	1.0 (9)	C11—C12—C16—O8	176.0 (6)
C2—C3—C4—C8	179.9 (5)	C13—C12—C16—O7	177.5 (6)
C3—C4—C5—C6	0.5 (9)	C11—C12—C16—O7	−3.1 (9)
C8—C4—C5—C6	−178.5 (5)	N1—N2—C17—N3	0.2 (7)
C4—C5—C6—C7	−1.5 (8)	C18—N3—C17—N2	−0.7 (7)
C4—C5—C6—I1	−179.1 (4)	N2—N1—C18—N3	−0.8 (7)
C5—C6—C7—C2	1.1 (9)	C19—N1—C18—N3	−174.9 (5)
I1—C6—C7—C2	178.7 (4)	C17—N3—C18—N1	0.9 (7)
C3—C2—C7—C6	0.4 (9)	C18—N1—C19—C20	105.3 (6)
C1—C2—C7—C6	−178.0 (5)	N2—N1—C19—C20	−68.3 (6)
C3—C4—C8—O4	177.5 (6)	N1—C19—C20—C21	144.5 (5)
C5—C4—C8—O4	−3.6 (10)	N1—C19—C20—C25	−37.4 (7)
C3—C4—C8—O3	−2.6 (8)	C25—C20—C21—C22	2.8 (9)
C5—C4—C8—O3	176.3 (6)	C19—C20—C21—C22	−179.0 (5)
O6—C9—C10—C11	−3.3 (10)	C20—C21—C22—C23	0.2 (9)
O5—C9—C10—C11	175.3 (6)	C21—C22—C23—C24	−2.2 (8)
O6—C9—C10—C15	177.7 (6)	C21—C22—C23—C23^i^	177.2 (6)
O5—C9—C10—C15	−3.6 (9)	C22—C23—C24—C25	1.3 (9)
C15—C10—C11—C12	0.6 (9)	C23^i^—C23—C24—C25	−178.2 (6)
C9—C10—C11—C12	−178.4 (5)	C21—C20—C25—C24	−3.7 (9)
C10—C11—C12—C13	−0.1 (9)	C19—C20—C25—C24	178.1 (6)
C10—C11—C12—C16	−179.5 (5)	C23—C24—C25—C20	1.7 (9)
C11—C12—C13—C14	−1.0 (9)		

Symmetry code: (i) −*x*, −*y*+2, −*z*+1.

### Hydrogen-bond geometry (Å, º)

**Table:**

*D*—H···*A*	*D*—H	H···*A*	*D*···*A*	*D*—H···*A*
O1—H1···O4^ii^	0.84	1.90	2.666 (6)	150
O3—H2···N3	0.84	1.84	2.642 (6)	159
O5—H3···O8^iii^	0.84	1.93	2.628 (6)	140
O1w—H5···O6^ii^	0.84	1.97	2.803 (7)	172
O1w—H6···O7	0.84	1.81	2.638 (7)	171
N2—H4···O1w	0.88	2.12	2.899 (6)	148

Symmetry codes: (ii) *x*+1, *y*−1, *z*; (iii) *x*−1, *y*+1, *z*.
